# Magnetization Transfer Ratio in Lower Limbs of Late Onset Pompe Patients Correlates With Intramuscular Fat Fraction and Muscle Function Tests

**DOI:** 10.3389/fneur.2021.634766

**Published:** 2021-03-16

**Authors:** Claudia Nuñez-Peralta, Paula Montesinos, Alicia Alonso-Jiménez, Jorge Alonso-Pérez, David Reyes-Leiva, Javier Sánchez-González, Jaume Llauger-Roselló, Sonia Segovia, Izaskun Belmonte, Irene Pedrosa, Antonio Martínez-Noguera, Briano Matellini-Mosca, Glenn Walter, Jordi Díaz-Manera

**Affiliations:** ^1^Radiology Department, Hospital de la Santa Creu i Sant Pau, Barcelona, Spain; ^2^Departament de Medicina, Universidad Autónoma de Barcelona, Barcelona, Spain; ^3^Philips Healthcare Iberia, Madrid, Spain; ^4^Neuromuscular Reference Center, Neurology Department, University Hospital of Antwerp, Edegem, Belgium; ^5^Neuromuscular Disorders Unit, Neurology Department, Hospital de la Santa Creu i Sant Pau, Barcelona, Spain; ^6^Centro de Investigación Biomédica en Red de Enfermedades Raras (CIBERER), Madrid, Spain; ^7^Rehabilitation Department, Hospital de la Santa Creu i Sant Pau, Barcelona, Spain; ^8^Department of Physiology and Functional Genomics, University of Florida, Gainesville, FL, United States; ^9^John Walton Muscular Dystrophy Research Center, Newcastle University, Newcastle upon Tyne, United Kingdom

**Keywords:** late onset Pompe disease, lower limb muscle, magnetic transfer ratio, intramuscular fat fraction, muscle function tests

## Abstract

**Objectives:** Magnetization transfer (MT) imaging exploits the interaction between bulk water protons and protons contained in macromolecules to induce signal changes through a special radiofrequency pulse. MT detects muscle damage in patients with neuromuscular conditions, such as limb-girdle muscular dystrophies or Charcot-Marie-Tooth disease, which are characterized by progressive fiber loss and replacement by fatty tissue. In Pompe disease, in which there is, in addition, an accumulation of glycogen inside the muscle fibers, MT has not been tested yet. Our aim is to estimate MT ratio (MTR) in the skeletal muscle of these patients and correlate it with intramuscular fat fraction (FF) and results of muscle function tests.

**Methods:** We obtained two-point axial Dixon and Dixon-MT sequences of the right thigh on a 1.5 Teslas MRI scanner in 60 individuals, including 29 late onset Pompe disease patients, 2 patients with McArdle disease, and 29 age and sex matched healthy controls. FF and MTR were estimated. Muscle function using several muscle function tests, including quantification of muscle strength, timed test quality of life scales, conventional spirometry obtaining forced vital capacity while sitting and in the supine position, were assessed in all patients.

**Results:** MTR was significantly lower in Pompe patients compared with controls (45.5 ± 8.5 vs. 51.7 ± 2.3, Student *T*-test, *p* < 0.05). There was a negative correlation between the MTR and FF muscles studied (correlation coefficient: −0.65, Spearman test: *p* < 0.05). MTR correlated with most of the muscle function test results. We analyzed if there was any difference in MTR values between Pompe patients and healthy controls in those muscles that did not have an increase in fat, a measure that could be related to the presence of glycogen in skeletal muscles, but we did not identify significant differences except in the adductor magnus muscle (48.4 ± 3.6 in Pompe vs. 51 ± 1.3 in healthy controls, Student *T*-test = 0.023).

**Conclusions:** MTR is a sensitive tool to identify muscle loss in patients with Pompe disease and shows a good correlation with muscle function tests. Therefore, the MT technique can be useful in monitoring muscle degeneration in Pompe disease in clinical trials or natural history studies.

## Introduction

Magnetization transfer (MT) imaging is a magnetic resonance (MRI) technique that exploits the magnetization exchange between water and tissue protons present in different environments. In normal tissue, hydrogen protons are present in two compartments: the so-called “free pool” with mobile protons in free water and a second compartment called the “bound pool,” consisting of protons bound to proteins and other macromolecules, such as glycogen (1). Protons within the free pool are responsible for the conventional MRI signal because they have a long and easily detectable T2 signal; on the other hand, protons bound to macromolecules have short T2 values due to their highly restricted motion and are not detected in routine MRI.

The interaction between these two compartments can be probed by measuring the exchange of energy from the bound to the free pool of protons. This is achieved by applying a special radiofrequency (RF) pulse called MT pulse (a low-power RF saturation pulse) that gives origin to the MT effect (2). The MT effect can be used to create additional contrast (MTC) in different tissues, and it is widely used in MR angiography, enhancing the representation of smaller peripheral branches of the vessels (3), or in multiple sclerosis in which the background suppression improves detection of acute lesions (4). The MT effect can also be quantified obtaining the magnetization transfer ratio (MTR), which provides insight into relaxation and exchange rates of free water and macromolecules (5).

Musculoskeletal tissue displays a pronounced MT effect, and it is already demonstrated to be a sensitive measure of muscle damage in patients with neuromuscular conditions, such as limb-girdle muscular dystrophies (LGMD) or Charcot-Marie-Tooth disease, which are disorders characterized by progressive muscle fiber loss and replacement by fatty tissue (6, 7). Nevertheless, the utility of MT has not been tested in Pompe disease yet, a disease in which there is an accumulation of glycogen within muscle fibers leading to cell death and replacement by fat. Estimation of the intramuscular fat fraction (FF) using the Dixon technique in Pompe patients is a useful biomarker for the follow-up of patients as we demonstrate that there is a progressive accumulation of fat in the skeletal muscles that precedes changes in muscle function tests in patients that have already started enzyme replacement therapy (8). However, presymptomatic patients do not always show an increase in fat accumulation even if they develop muscle weakness, suggesting that the identification of changes related to glycogen accumulation could be useful to monitor progression of the disease, especially in these presymptomatic cases. Our hypothesis is that MT could identify early structural changes related to the accumulation of glycogen before degeneration of the muscle fibers to substitution by fatty tissue takes place in the muscles. To confirm our hypothesis, we studied a cohort of patients with late-onset Pompe disease (LOPD) using the MT technique and compared the results with those obtained in healthy controls. Additionally, we correlated MTR values with intramuscular FF and muscle function tests.

## Methods

### Study Design and Participants

This is a transversal cross-sectional study involving 60 individuals who were studied at the Hospital de la Santa Creu i Sant Pau (HSCSP) in Barcelona. The study is registered in *ClinicalTrials.gov* with the identifier NCT01914536. The HSCSP ethics committee approved the study, and all participants signed an informed consent form. All study procedures were performed in accordance with Spanish regulations.

The inclusion criteria for the study were (1) genetically confirmed diagnosis of Pompe disease or McArdle disease; (2) no contraindications to MRI; and (3) willingness to complete all muscle function tests, respiratory assessment, and patient-reported outcomes measures. As control, we included a group of healthy volunteers age- and sex-matched with the Pompe patients. We ruled out other neuromuscular conditions in all participants in the study based on clinical data and results of complementary tests, such as blood analysis, spinal MRI, or EMG when needed.

All patients were studied by three physiotherapists with long experience in neuromuscular disorders at HSCSP. The physiotherapists evaluated muscle function using the following tests: the 6-min walking test, time to walk 10 m, timed up-and-go test, time to climb up and down four steps, and the Motor Function Measure 20-item scale (MFM-20). All timed tests were performed asking the patient to not use aids for walking. Muscle strength was studied using both the Medical Research Council (MRC) scale and hand-held myometry. Daily life activities were studied using the activity limitations scale for patients with upper and/or lower limb impairments, and quality of life was analyzed using both the Individualized Neuromuscular Quality of Life Questionnaire and the Short Form 36 questionnaire. We obtained forced vital capacity (FVC), both seated and lying down, using the Carefusion Microlab ML 3500 MK8 spirometer (Carefusion, Yorba Linda, CA, USA). These last two tests were added due their being commonly used in Pompe disease patients to measure their clinical status (9). Finally, Creatine kinase levels in serum were quantified in the HSCSP biochemistry laboratory following standard protocols.

### MRI

All patients were examined in a 1.5 MR system (1.5 Achieva dStream; Philips, Best, NL) at HSCSP in the supine position with stretched legs using a 32-channel body coil. Axial 3-D Dixon FFE on the middle third of both thighs was performed with the following parameters: TR/TE1/TE2 = 5.78/1.8/4 ms, flip angle = 15, FOV = 520 × 340 × 300, acquired voxel size = 1 × 1 × 3 mm^3^; acquisition time was 2 min 33 s. Also, another 3-D Dixon FFE sequence was acquired with and without an off-resonance magnetization transfer contrast prepulse active, TR/TE1/TE2 = 32/1.8/4 ms, flip angle = 15, FOV = 400 × 200 × 114 mm, acquired voxel size = 1 × 1 × 3 mm^3^; total acquisition time for images with and without MTC module active was 7 min 58 s.

### Analysis

**Figure 1:**
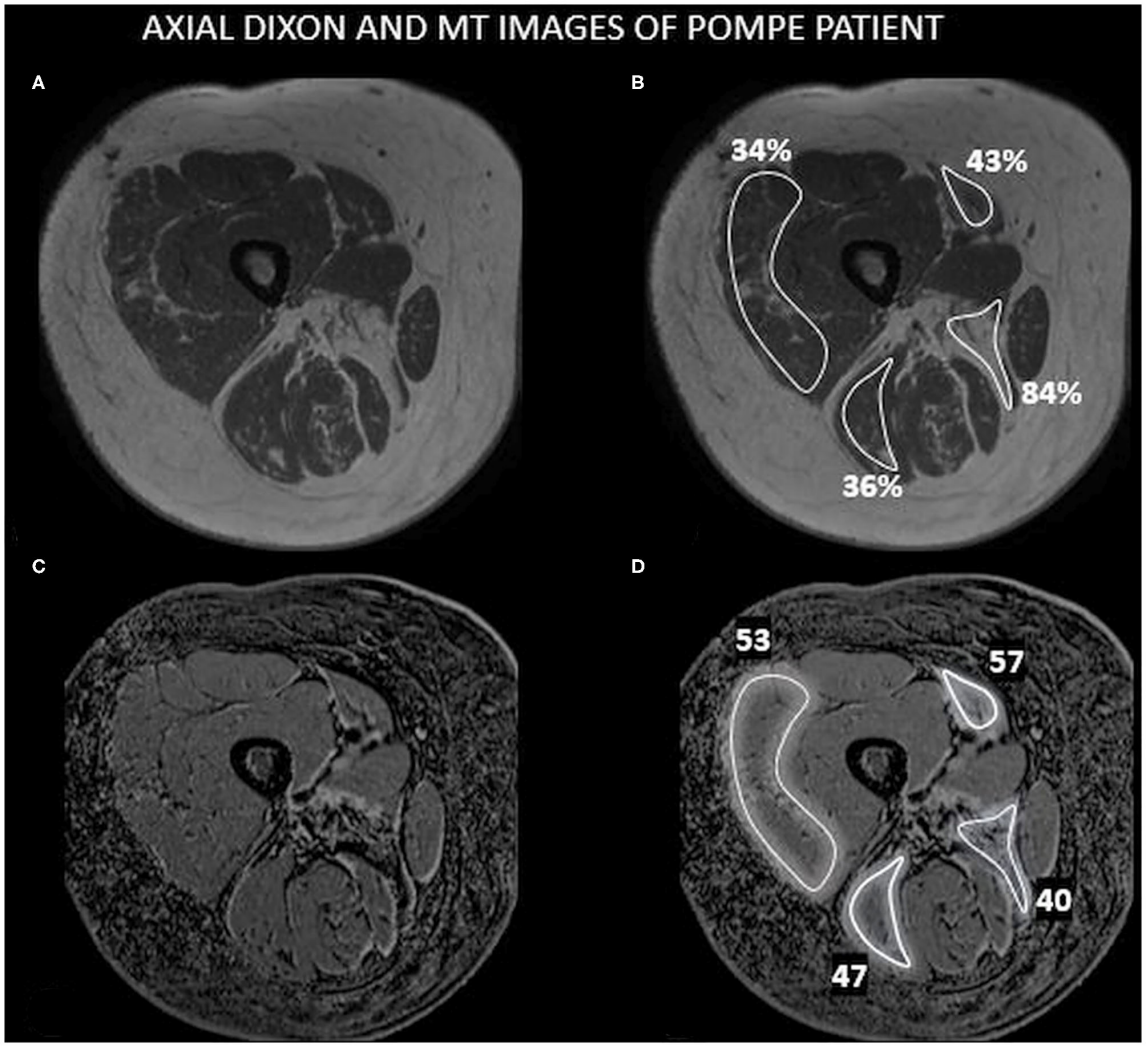
An example of Dixon and MT images. This figure shows an axial image of the right medial thigh of a Pompe patient including the muscles studied in this paper with the ROIs drawn and the values obtained for FF and MTR. **(A)** 3-D Dixon imaging of the thigh. **(B)** ROIs drawn in **(A)** showing the results of FF observed. **(C)** Dixon-MTC imaging of the thigh. **(D)** ROIs drawn in **(C)** showing the results of MTR.

**Figure 2:**
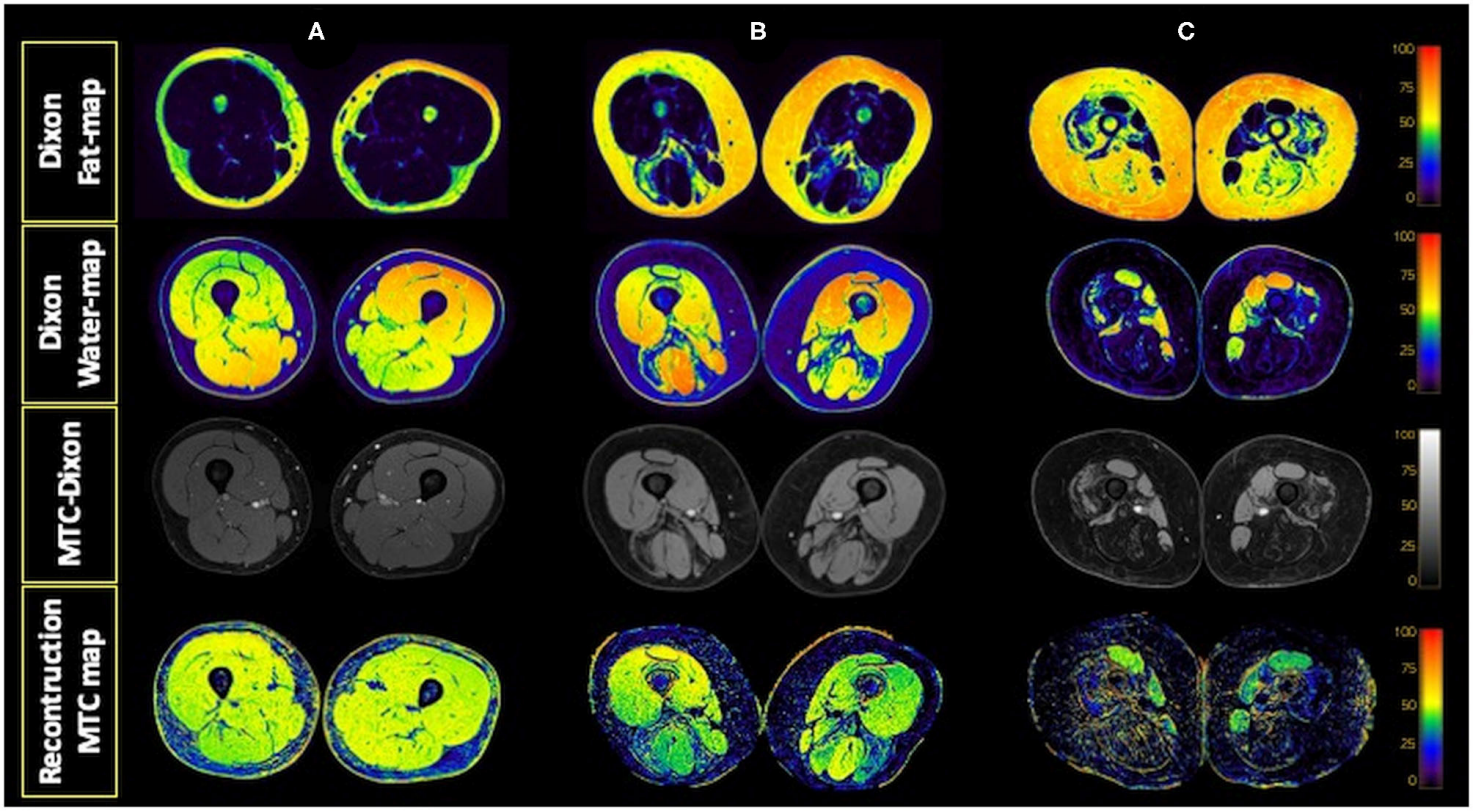
Dixon water and fat maps and MTC-Dixon images obtained. Examples of fat and water maps generated from the data obtained with the Dixon sequence and of the images obtained with the MTC-Dixon sequence and the map generated. Axial sections of the thighs of a control **(A)** and two patients with Pompe disease, one with moderate involvement **(B)** and the other with severe muscle involvement **(C)**.

We analyzed both sequences in the middle right thigh of all patients. Regions of interest (ROIs) were manually drawn by three investigators on one slice of the following muscles: vastus lateralis (VL), sartorius (Sar), the long head of the biceps femoris (BLH), and adductor magnus (AM), keeping a reasonable distance from the fascia and subcutaneous fat tissue. FF was obtained using a Philips Research Image Development Environment (PRIDE) tool developed for this purpose. MTR was calculated using the following formula in which Mo and MSat refer to the images without and with the saturation prepulse. MTR values were expressed in percentage. Figure 1 shows an example of MT imaging, and the ROIs drawn and Figure 2 shows an example of the images obtained in this study.

(1) $$\begin{array}{r} MTR\;=\left(\;\frac{Mo\;-\;M\;Sat}{Mo}\right)\; \end{array}$$

### Statistics

We confirmed that the variables to be analyzed were normally distributed using a Kolmogorov–Smirnov test, and therefore, parametric statistical studies were used. We used the Student *T*-test to analyze if the differences observed in FF and MTR between Pompe and controls were statistically significant. We used the Pearson test to study if correlations between variables were significant. We consider that a correlation was good if the correlation coefficient was higher than 0.6. The significance level for all statistical studies was set at *p* < 0.05, and *post-hoc* Bonferroni corrections were used when needed. Statistical studies were performed with SPSS for Mac computers (Version 21, SPSS Inc., Chicago, IL).

## Results

### Patients Included

We included in the study 29 LOPD patients of which 23 patients were symptomatic and treated with enzymatic replacement therapy (ERT) and 6 patients were presymptomatic and not treated with ERT. Clinical features of LOPD patients included in this study have been reported before (10). We also included in the study 2 patients with McArdle disease without muscle weakness at clinical examination and 29 healthy age- and sex-matched controls. Main demographic and clinical data of the individuals included in the study are summarized in Table 1.

**Table 1:** Mean demographic and clinical data of the patients included in the study.

	**Pompe**	**McArdle**	**Controls**
Individuals (*n*)	29	2	29
Age at MRI	41.2 ± 21.3 y.o.	44 and 38 y.o	45.2 ± 22.7 y.o.
Use of ERT (*n*)	21		
Aids for walking (*n*)	12	–	–
Need of ventilation (*n*)	11	–	–

*MRI, magnetic resonance imaging; ERT, enzymatic replacement therapy; N, number of patients*.

### MRI Results

**Figure 3:**
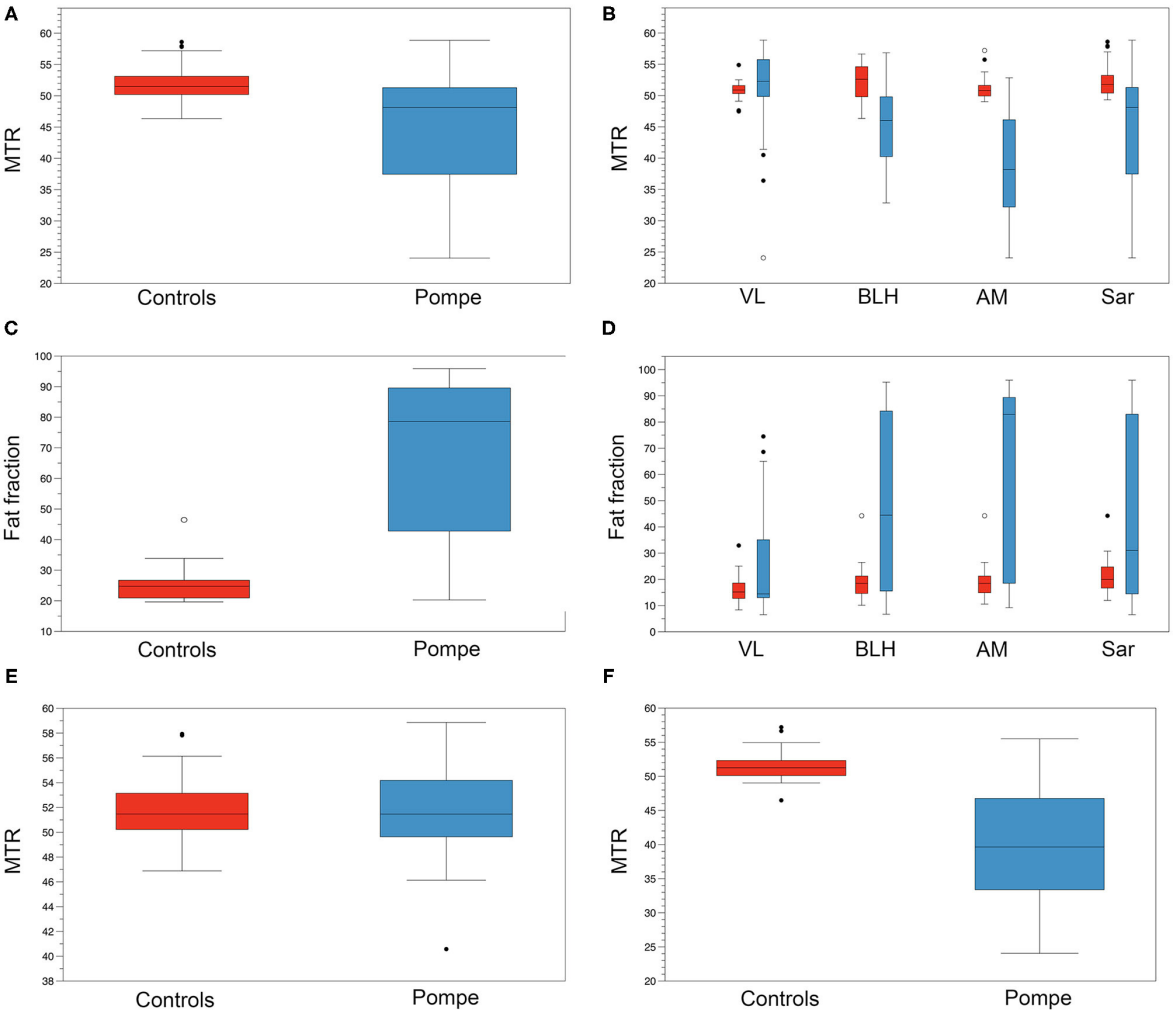
MTR and FF in controls and Pompe patients. **(A)** Mean thigh MTR value in controls (red) and Pompe patients (blue). **(B)** MTR value of VL, BLH, AM, and Sar in controls (red) and Pompe patients (blue). **(C)** Mean thigh FF value in controls (red) and Pompe patients (blue). **(D)** FF of VL, BLH, AM, and Sar in controls (red) and Pompe patients (blue). **(E)** Mean thigh MTR value in individuals with mean thigh FF lower than 20% in controls (red) and Pompe patients (blue). **(F)** Mean thigh MTR value in individuals with mean thigh FF higher than 20% in controls (red) and Pompe patients (blue). Mean value and standard deviation are shown.

We found lower MTR average value in LOPD patients' muscles (45.5 ± 8.5) compared with controls (51.7 ± 2.3) (Student *T*-test, *p* < 0.001; Figure 3A). When analyzed separately, we identified significant lower values in AM and BLH but not in VL nor Sar in LOPD patients compared with controls (Figure 3B). MTR average value was lower in symptomatic compared with presymptomatic (41.9 ± 9.8 vs. 51 ± 4, respectively, Student *T*-test *p* < 0.001). There were no differences in MTR between controls and presymptomatic patients.

We found higher mean thigh FF in LOPD patients' muscles (44.9 ± 32.8%) compared with controls (17.86 ± 6.8%) (Student *T*-test, *p* < 0.001; Figure 3C). When analyzed separately, we identified higher values in AM, BLH, and VL but not in Sar in LOPD patients compared with controls (Figure 3D). Mean thigh FF was significantly higher in symptomatic compared with presymptomatic LOPD patients (32.6 ± 7.7 vs. 21.24 ± 7.3, respectively, Student *T-*test *p* < 0.001). There were no differences in mean thigh FF between controls and presymptomatic patients.

**Figure 4:**
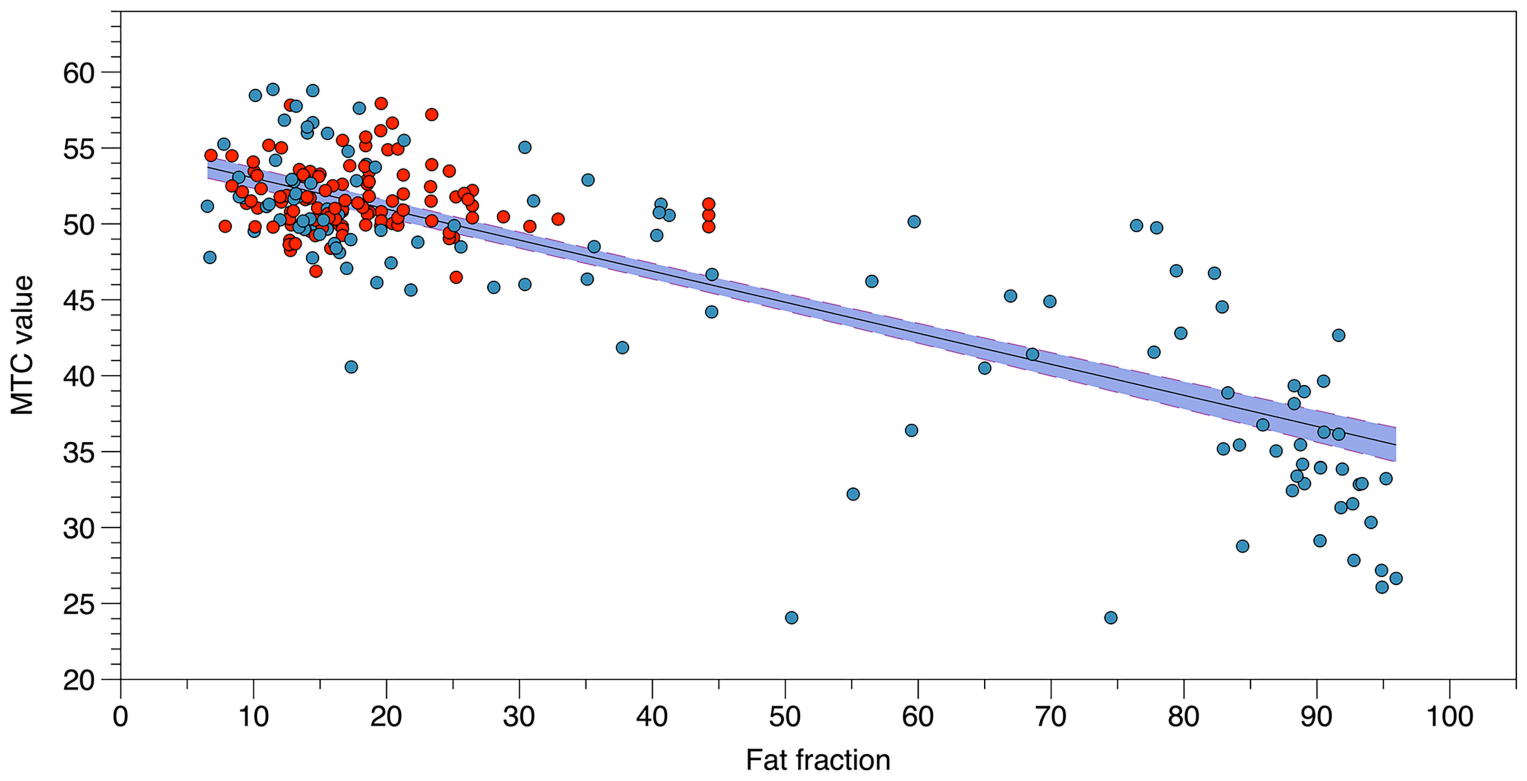
Correlation between MTR value and FF. The figure shows the correlation between MTR value and FF in each muscle analyzed from controls (red) and patients with Pompe disease (blue).

We did not identify significant differences in MTR in those muscles with FF lower than 20% between LOPD and healthy controls when the four individual muscles were analyzed together (Figures 3E,F). However, when analyzed separately, we observed significant differences in MTR in the AM (48.4 ± 3.6 in Pompe vs. 51 ± 1.3 in controls, Student *T*-test = 0.023). We observed a negative correlation between MTR value and FF in muscles studied (correlation coefficient: −0.65, Spearman test *p* < 0.0001; Figure 4).

### Correlation With Results of the Muscle Function Tests

We observed a significant correlation between mean thigh MTR value and the results of muscle function tests, including timed tests, such as the 6MWT or the time to walk 10 m, and measures of muscle strength, such as the composite MT value of the lower limbs. Moreover, we identified a good correlation between individual muscle MTR values and specific muscle assessments as is shown in Table 2.

**Table 2:** Correlation between MTC values and results of the muscle function tests.

**Mean MTC value**		
	**Correlation coefficient**	**Significance**
Time to walk 10 m	−0.72	0.0001
6 MWT	0.813	0.0001
Time up go	−0.78	0.0001
Time climb up 4 Steps	−0.79	0.0001
Time go down 4 Steps	−0.75	0.0001
MRC lower limbs	0.71	0.0001
Activlim	0.59	0.001
CK levels	0.12	0.71
**MTC adductor magnus**		
Hip adduction	0.63	0.0001
**MTC biceps long head**		
Knee flexion	0.54	0.003
**MTC vastus lateralis**		
Knee extension	0.38	0.52

*6MWT, 6 min walking Test. Pearson test was performed, and p-values are shown*.

## Discussion

We observed that MT is an indirect measurement of muscle loss in LOPD patients, and it correlates with results of different muscle function tests commonly used in clinical trials and natural history studies. We also identified a good correlation between muscle FF and muscle MTR value, suggesting that MTR values decrease in relation to progressive loss of muscle fibers that are replaced by fatty tissue.

Quantitative muscle MRI is progressively being implemented in the follow-up of patients with neuromuscular diseases, including Pompe disease, in clinical trials and natural history studies (11–13). Dixon imaging allows calculating the FF, which is the amount of skeletal muscle replaced by fatty tissue. FF is shown to correlate with the results of muscle function tests in different diseases, including but not limited to Pompe disease. Moreover, Dixon has proved to be useful to follow up the progression of fat replacement in LOPD patients in longitudinal studies. Previous studies have identified risk factors associated with FF, such as early age of onset of symptoms and disease duration (8).

Based on these results, Dixon sequences are being implemented in the new clinical trials that are being designed in this disease. MT imaging is based on the magnetization exchange between protons present in the free-water compartment and protons bound to macromolecules. The more hydrated the macromolecules are, the more magnetization transfer is obtained. MT from fat is low as it is a hydrophobic tissue. Hydrophobic properties of lipids cause them to experience limited MT; for that reason, MT is generally low in tissues with high fat content (14–16). However, it is known that fat generates chemical shift artifacts that could compromise the characterization and analysis of tissue. The use of traditional fat-suppression techniques in MT studies can affect the MTR measurements, but the use of water images obtained from Dixon sequences avoids possible confounding effects derived from the fat signal for MTR calculation (17, 18). For these reasons, in this study, we decided to use a two-point Dixon sequence with an off-resonance prepulse. However, the signal obtained in muscles replaced by fat could also be influenced by other concomitant components, such as fibrotic tissue or inflammation. Therefore, MTR is not directly measuring only fat replacement, but in our opinion, is an indirect measurement of loss of muscle fibers that reduce the values obtained. In this sense, our results are similar to the ones reported in other neuromuscular diseases, such as LGMD, inclusion body myositis, Charcot-Marie-Tooth disease, or spinal muscle atrophy (6, 7). We observed lower MTR values in symptomatic patients compared with non-symptomatic patients and controls. To fully understand the biophysical origins of MT in Pompe muscle, a more detailed quantitative MT study is warranted that takes into account pool sizes, T1, lipids, and multiple transfer magnetization transfer mechanisms (5, 19).

**Figure 5:**
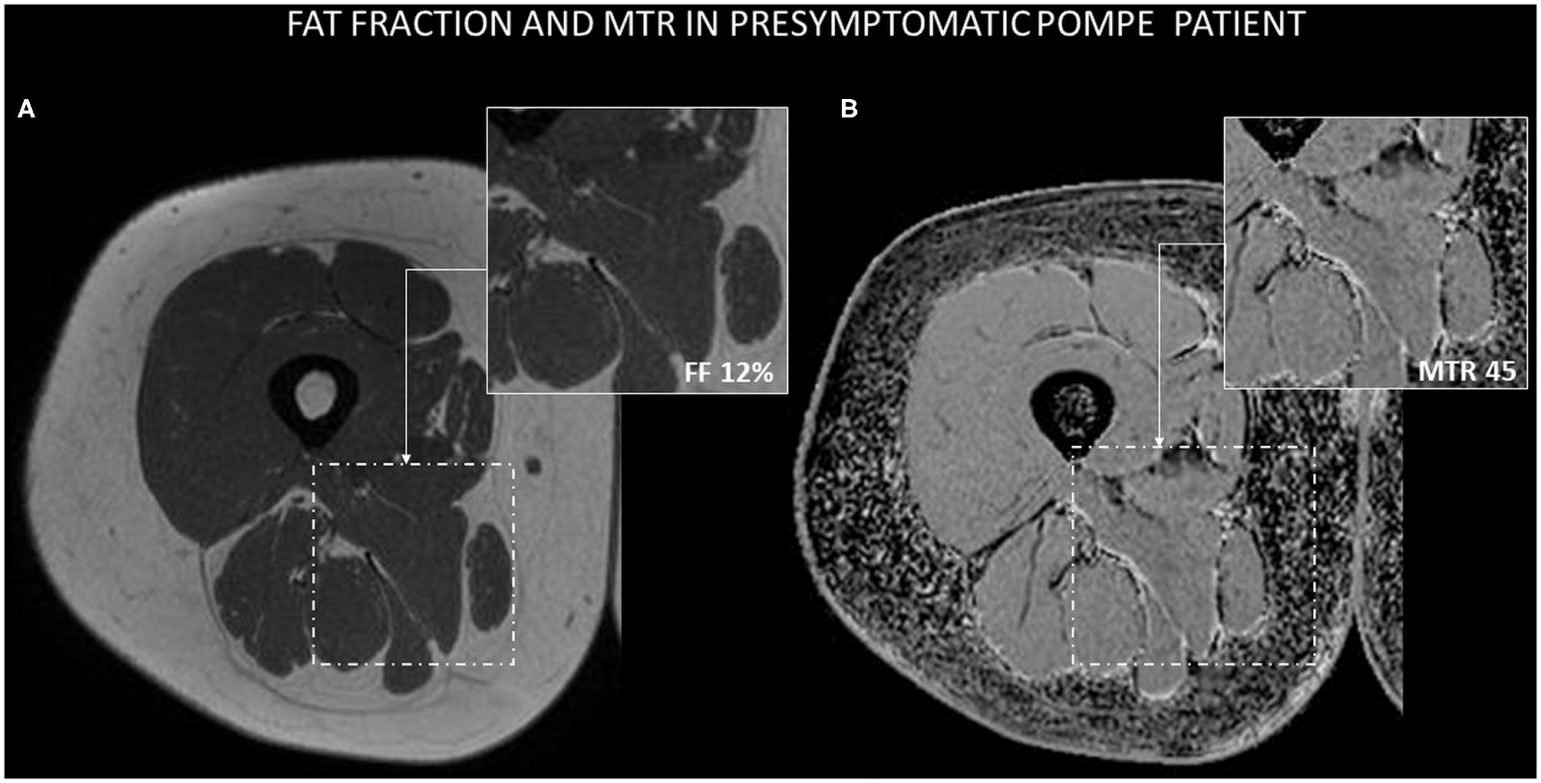
Example of Dixon and MTR images in a presymptomatic Pompe patient. **(A)** AM muscle is not macroscopically infiltrated by fat, and the estimation of FF using the Dixon technique is 12%. **(B)** MTR value in AM is 45.

Our initial hypothesis was that MT could be useful as an indirect measurement of glycogen. Glycogen is a highly hydrated molecule, and therefore, it was tempting to hypothesize that, in patients with Pompe disease who accumulate glycogen in their muscles, MTR could increase because of the accumulation of water bound to the glycogen. In this study, we observed that fat replacement is a confounding factor because it reduces the MTR signal considerably and makes difficult the identification of the effect of other tissue components on MTR. However, MT values in muscles with low levels of fat replacement (<20% FF) are not statistically different between healthy controls and LOPD patients except for the AM. Additionally, we have not seen differences between presymptomatic non-treated and symptomatic treated patients in the MTR values in muscles with FF lower than 20% (Figure 5). There are two potential explanations for our findings. On one hand, it is possible that the amount of glycogen accumulated in these muscles was too low to induce any change in MTR. It is well-known that LOPD patients accumulate less glycogen than infantile onset patients (IOPD). Muscle biopsies of LODP patients can show mild accumulation of glycogen or even be normal, and muscle biopsies in IOPD patients are characterized by a massive accumulation of glycogen. On the other hand and based on the physiopathology of the disease, it is probable that the accumulation of glycogen was more prominent in those muscles undergoing fatty replacement, but if that was the case, MTR could be influenced by the loss of muscle fibers and expansion of fat, producing, as a result, a decrease in MTR values (20–22). We have also tested MT in 2 patients with glucogenosis type V, usually known as McArdle disease, who had mean thigh FF lower than 10%, and we have not seen any change in MTR values compared with controls. In our opinion, to completely rule out MTR as an indirect measure of glycogen in patients, we should explore it in pretreated IOPD patients because they accumulate larger amounts of glycogen in their muscles.

Interestingly, we have seen a statistically significant decrease in MTR values in the AM muscle in presymptomatic patients without an increase in FF. We previously identified that this muscle is one of the muscles earlier replaced by fat in the progression of the disease (8). Two of these patients developed mild hip adduction weakness soon after being scanned in this study. In our opinion, this result probably reflects the loss of muscle fibers that could be the cause of the weakness rather than accumulation of glycogen as we would expect an increase in the MTR values. It is possible that MT has high sensitivity to detect mild muscle fiber loss even when these fibers have not been replaced by fat yet, and Dixon studies do not detect an increase in fatty tissue. Accordingly, McDaniel et al. reported a decrease in MTR value in muscles of patients with muscular dystrophies that were not replaced by fat, which could be related, in our opinion, to the existence of early muscle damage not yet leading to fat replacement.

The main limitation of our study is that we have not used a gold standard test to study the amount of glycogen present in the muscles of the patients, such as a muscle biopsy or specific imaging sequences able to identify glycogen, such as 13C and 1H spectroscopy (23), chemical exchange saturation transfer imaging of glycogen (GlycoCEST) (24–26), and the recently described nuclear Overhauser enhancement of glycogen (Glyco-nOe) (27, 28). All these sequences are able to identify glycogen in muscles and/or liver but are not available in conventional hospitals not focused on research. Some of the advantages of the MT technique are that it is available in many hospitals, does not require a specific coil, and the acquisition is relatively fast and easy to analyze.

We have seen that MT values correlate with FF and, therefore, could be used to monitor disease progression over time although this should be explored in future longitudinal studies. The only longitudinal study published so far shows that MT measurements decreased in Charcot-Marie-Tooth and IBM patients after 1 year of follow-up in relation to the accumulation of fatty tissue identified using Dixon (29).

## Conclusion

The MTR estimation constitutes a sensitive tool for the identification of cellular damage in patients with Pompe disease. It is correlated with muscle loss and muscle function tests. Therefore, MT should be further explored as a tool for monitoring muscle degeneration in Pompe disease in clinical trials or natural history studies.

## Data Availability Statement

The original contributions generated for the study are included in the article/supplementary material, further inquiries can be directed to the corresponding author/s.

## Ethics Statement

The studies involving human participants were reviewed and approved by the Ethics Committee of the Hospital de la Santa Creu i Sant Pau, Barcelona. Written informed consent to participate in this study was provided by the participants' legal guardian/next of kin.

## Author Contributions

CN-P and PM: concept and design of the study, acquisition and analysis of data, and drafting the paper. AA-J and JA-P: acquisition and analysis of data, and drafting the paper. DR-L, JL-R, IB, IP, and BM-M: concept and design of the study, acquisition of data, and drafting the paper. JS-G, AM-N and GW: concept and design of the study, and drafting the paper. SS: concept and design of the study, coordination of the study, and drafting the paper. JD-M: concept and design of the study, obtaining funding for the study, acquisition and analysis of data, and drafting the paper. All authors contributed to the article and approved the submitted version.

## Conflict of Interest

PM was employed by company Philips Healthcare Iberia. The remaining authors declare that the research was conducted in the absence of any commercial or financial relationships that could be construed as a potential conflict of interest.
