# Corrigendum: Magnetization Transfer Ratio in Lower Limbs of Late Onset Pompe Patients Correlates With Intramuscular Fat Fraction and Muscle Function Tests

**DOI:** 10.3389/fneur.2021.727020

**Published:** 2021-07-13

**Authors:** Claudia Nuñez-Peralta, Paula Montesinos, Alicia Alonso-Jiménez, Jorge Alonso-Pérez, David Reyes-Leiva, Javier Sánchez-González, Jaume Llauger-Roselló, Sonia Segovia, Izaskun Belmonte, Irene Pedrosa, Antonio Martínez-Noguera, Briano Matellini-Mosca, Glenn Walter, Jordi Díaz-Manera

**Affiliations:** ^1^Radiology Department, Hospital de la Santa Creu i Sant Pau, Barcelona, Spain; ^2^Departament de Medicina, Universidad Autónoma de Barcelona, Barcelona, Spain; ^3^Philips Healthcare Iberia, Madrid, Spain; ^4^Neuromuscular Reference Center, Neurology Department, University Hospital of Antwerp, Edegem, Belgium; ^5^Neuromuscular Disorders Unit, Neurology Department, Hospital de la Santa Creu i Sant Pau, Barcelona, Spain; ^6^Centro de Investigación Biomédica en Red de Enfermedades Raras (CIBERER), Madrid, Spain; ^7^Rehabilitation Department, Hospital de la Santa Creu i Sant Pau, Barcelona, Spain; ^8^Department of Physiology and Functional Genomics, University of Florida, Gainesville, FL, United States; ^9^John Walton Muscular Dystrophy Research Center, Newcastle University, Newcastle upon Tyne, United Kingdom

**Keywords:** late onset Pompe disease, lower limb muscle, magnetic transfer ratio, intramuscular fat fraction, muscle function tests

In the published article, there was an error regarding the affiliation(s) for Claudia Nuñez-Peralta. As well as having affiliation(s) ^**^ 1 ^**^, they should also have ^**^2Departament de Medicina, Universidad Autónoma de Barcelona, Barcelona, Spain^**^.

The authors apologize for this error and state that this does not change the scientific conclusions of the article in any way. The original article has been updated.

